# Does food responsiveness change in people with first-episode psychosis over a period of 3 months after commencing antipsychotics? Preliminary results

**DOI:** 10.1097/XCE.0000000000000274

**Published:** 2022-12-08

**Authors:** Adrian H. Heald, Mark Shakespeare, Adrian Phillipson, Janet Cade, Petra Netter, Suzanne Higgs

**Affiliations:** a Department of Diabetes and Endocrinology, Salford Royal Hospital, Salford; b The School of Medicine and Manchester Academic Health Sciences Centre, University of Manchester; c Rotherham, Doncaster and South Humber (RDASH) NHS Foundation Trust; d Nutritional Epidemiology Group, School of Food Science and Nutrition, University of Leeds, Leeds; e Department of Psychology, University of Giessen, Germany; f School of Psychology, College of Life and Environmental Sciences, University of Birmingham, UK

Weight gain occurs in many patients with schizophrenia [1], a diagnosis often subsequently made in individuals who first present with psychosis (characterized by culture-dyscongruent beliefs, disordered thinking and abnormal experiences). Frequently weight gain occurs in the first few weeks and months after diagnosis [2] and is related to an increase in appetite and food intake, with often double portions consumed and high-calorie snacks taken between meals. Early weight gain is a predictor of longer-term weight gain [3]. Weight gain in psychosis is associated with an increased likelihood of the development of type 2 diabetes [4] and increased cardiovascular risk with associated shortened life expectancy [5].

Many of the drugs that are effective in treating psychosis are associated with changes in the way that people experience reward when they eat [6].

To date, there has been little research in this area [7], and as such little is known about people’s response to food and appetite shortly after commencing antipsychotic medication.

The purpose of this project was to increase our understanding of exactly what are the drivers of weight gain in terms of an individual’s experience of food reward and reduced satiety – and therefore how we can help people with schizophrenia to keep their weight down. At this stage, we are looking at the feasibility of applying currently available evaluation tools to people in this situation.

Our aim was to determine if food responsiveness changes in people with first-episode psychosis (FEP) over a period of 3–6 months after commencing antipsychotics. The study was approved by the West Midlands Ethics Committee (Reference 20/WM/0146).

A convenience sample was used with the aim of recruiting service users from early intervention services. This was designed as a feasibility study to provide data to underpin a fully powered, larger investigation.

The study was conducted in early intervention services across the Rotherham, Doncaster and South Humber (RDASH) National Health Service Foundation Trust. Early intervention services provide support and treatment for people with FEP in England. Participation in the study was offered to those who had commenced antipsychotic medication within the previous 6 weeks, in the context of a diagnosis of FEP.

Inclusion criteria: patients diagnosed with FEP, aged 18–35 years and attending the RDASH early intervention service.

Exclusion criteria were: age <18 years or >50 years, unable or unwilling to provide informed consent, pregnancy, non-English speaking, intellectual disability or communication difficulties hindering their ability to complete assessments or mental health or distress levels too high as deemed by their care coordinator.

## Rating scales applied were

Dutch Eating Behaviour Questionnaire (DEBQ) [8]: this measures restrained eating, emotional eating and external eating.

Intuitive Eating Scale [9]: this measures an individual’s tendency to follow their physical hunger and satiety cues.

Power of food questionnaire [10]: this measures responsiveness to the food environment.

The loss of control over eating scale [11]: this measures a global sense of whether individuals experience loss of control over eating.

The questionnaires were administered through a telephone interview at baseline and 3–6 months later.

In the event, only six participants were recruited because of severe acute respiratory syndrome coronavirus 2 (SARS‐CoV‐2) pandemic constraints on recruitment. The six participants (four men and two women) were aged between 17 and 31 years. All were diagnosed with FEP. Time from initiation of treatment with an antipsychotic to the first interview varied from 13 to 39 days.

Three people were started on olanzapine at the dose of 5 or 10 mg daily, one was started on risperidone at 1 mg daily and two were started on aripiprazole at 10 and 15 mg daily, respectively.

**Fig. 1:**
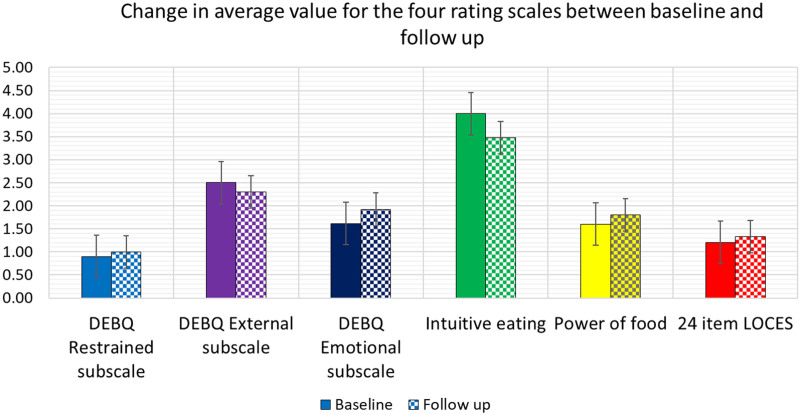
Change in DEBQ scores, intuitive eating, power of food and LOCES scales from baseline to 3- or 6-month follow-up interview. DEBQ, Dutch Eating Behaviour Questionnaire; LOCES, loss of control over eating scale.

Baseline scores are shown in Fig. 1. At the beginning of antipsychotic treatment, there was moderate responsiveness to food and a moderate tendency to follow hunger/satiety cues, with a low emotional drive to eat and low compulsion to eat.

At 3–6-month follow-up (pooled results) (Fig. 1), there was: a trend for a decrease in the DEBQ emotional eating score (*P* = 0.06) reflecting less response to external cues such as the aroma of food, presence of food or watching others eat and a decrease in the intuitive eating score (reflecting a lesser tendency to follow hunger/satiety cues) (*P* = 0.02).

Our preliminary results reveal that at the beginning of antipsychotic treatment there was moderate responsiveness to food and a moderate tendency to follow hunger/satiety cues, with a low emotional drive to eat and low compulsion to eat. At 3- to 6-month follow-up, there was a decreased response to satiety cues (intuitive eating score) and a trend for a decrease in the DEBQ emotional eating score (*P* = 0.06) reflecting less response to external cues. Externality theory describes external eating as eating in response to food-related stimuli, regardless of hunger or satiety [12]. The change to less responsiveness to external food-related stimuli may relate to the effects of the acute illness itself or the effects of antipsychotic treatment on response to food.

It is important to point to a recent report that even in the absence of antipsychotic medication relative to matched controls, patients with first-episode psychosis who were not taking antipsychotic medication consumed more saturated fat and showed an altered association between BMI and neural response to food cues [13]. Therefore what we have observed here is likely the consequence of multiple interacting factors including prescribed medication.

Although we had few participants and were not able to differentiate responses by antipsychotic agents, we feel that this is a relevant contribution to the literature given the limited work so far in this area [7]. These findings, although preliminary highlight how maladaptive eating patterns and alterations in the association between BMI and neural responses to food cues may be established early in the course of a psychotic illness which may be subsequently diagnosed as schizophrenia.

The difference between the baseline and the follow-up eating behavior scores may provide important clues as to the change in eating behavior with antipsychotic treatment. Greater knowledge of this will enable more effective targetting of interventions to reduce the likelihood of weight gain with its attendant consequences of elevation of cardiometabolic risk [4,5].

We accept that the numbers here are too small to draw any definite conclusions. The study was very much constrained by the recruiting period starting and completing in a period when face-to-face consultations in mental health services and interactions between healthcare professionals were occurring on a very limited basis. Furthermore, not all participants were contactable for the interview at 6 months so we pooled the 3-month results with the 6-month results where there was no 6-month follow-up. Nevertheless, we feel that our findings provide the basis for a future larger and more comprehensive study, which takes account of the practicalites of recruiting people who face the challenges of FEP.

## Acknowledgements

This study was funded by the University of Manchester N8 Pump Priming Fund. Grant Code: AA16858.

The data that support the findings of this study are available from the corresponding author upon reasonable request.

A.H. and A.P. led on the conceptualization, design and writing up of this project. M.S. conducted the data analysis and contributed to all aspects of the article. J.C. and S.H. advised on all aspects of the methods including the specific rating scales employed and also contributed to the Discussion. P.N. acted as a senior leader of the article.

### Conflicts of interest

There are no conflicts of interest.
